# Dietary Methionine Level Impacts the Growth, Nutrient Metabolism, Antioxidant Capacity and Immunity of the Chinese Mitten Crab (*Eriocheir sinensis*) under Chronic Heat Stress

**DOI:** 10.3390/antiox12010209

**Published:** 2023-01-16

**Authors:** Jiadai Liu, Cong Zhang, Xiaodan Wang, Xinyu Li, Qincheng Huang, Han Wang, Yixin Miao, Erchao Li, Jianguang Qin, Liqiao Chen

**Affiliations:** 1 Laboratory of Aquaculture Nutrition and Environmental Health, School of Life Sciences, East China Normal University, 500 Dongchuan Road, Shanghai 200241, China; 2 Key Laboratory of Tropical Hydrobiology and Biotechnology of Hainan Province, Hainan Aquaculture Breeding Engineering Research Center, College of Marine Sciences, Hainan University, Haikou 570228, China; 3 College of Science and Engineering, Flinders University, Adelaide, SA 5001, Australia

**Keywords:** dietary methionine level, chronic heat stress, nutrient metabolism, oxidative stress, antioxidant capacity, immunity, *Eriocheir sinensis*

## Abstract

This study examined whether diets with high dietary methionine levels could alleviate chronic heat stress in Chinese mitten crab *Eriocheir sinensis*. Crabs were fed three dietary methionine levels of 0.49%, 1.29% and 2.09% for six weeks. The analyzed methionine concentration of diets was 0.48%, 1.05% and 1.72%, respectively. Crabs were fed three different supplemental concentrations of dietary methionine at 24 °C and 30 °C, respectively. The trial was divided into six groups with five replicates in each group, and 40 juvenile crabs (initial average weight 0.71 ± 0.01 g) in each replicate. During the trial, crabs were fed twice daily (the diet of 4% of the body weight was delivered daily). The effects of dietary methionine level on nutrient metabolism, antioxidant capacity, apoptosis factors and immunity were evaluated at a normal water temperature of 24 °C and high temperature of 30 °C. Feed conversion ratio decreased under chronic heat stress. Chronic heat stress increased weight gain, specific growth rate, molting frequency, and protein efficiency ratio. The survival of crabs decreased under chronic heat stress, whereas a high level of dietary methionine significantly improved survival. Chronic heat stress induced lipid accumulation and protein content reduction. The high-methionine diet decreased lipid in the body and hepatopancreas, but increased protein in the body, muscle and hepatopancreas under chronic heat stress. Simultaneously, the high dietary methionine levels mitigated oxidative stress by reducing lipid peroxidation, restoring the antioxidant enzyme system, decreasing apoptosis and activating immune function under chronic heat stress. This study suggests that supplementing 1.72% dietary methionine could alleviate the adverse effects of a high water temperature in *E. sinensis* farming.

## 1. Introduction

Temperature is a key environmental factor affecting aquatic organism growth and survival [1]. In summer, the water temperature is usually higher than the optimal survival temperature for most aquatic animals, leading to an ecological deterioration of aquaculture waters and the frequent occurrence of aquatic animal diseases [2]. Several studies have reported that heat stress affects many aspects of aquatic animals, including growth, metabolism, antioxidant capacity, and immune function [3,4,5]. For example, heat stress caused growth inhibition and increased mortality in juvenile Atlantic salmon *Salmo salar* and spotted wolffish *Anarhichas minor* [6,7]. Similarly, tiger shrimp *Penaeus monodon*’s larval size and molting frequency rate (MR) decreased as the water temperature exceeded the optimum survival temperature [7,8,9]. However, the weight gain (WG) of the Chinese mitten crab (*Eriocheir sinensis*) increased under heat stress [10]. The adverse impact of heat stress is mainly caused by nutrient metabolism disorders and oxidative stress induced by reactive oxygen species (ROS) [3]. In general, animals need more energy consumption to adapt to heat stress [11]. However, heat stress usually inhibits lipid mobilization and promotes lipid synthesis and deposition, forcing protein catabolization for energy [12]. Excessive ROS by heat stress impairs the antioxidant system, leading to apoptosis and immunosuppression, and further destroying the biofilm system, cellular structure and function [13]. In naked carp (*Gymnocypris przewalskii*), acute heat stress significantly inhibited liver antioxidant enzymes and caused immune suppression by inhibiting the Toll-like receptor (*Tlrs*) pathway [14]. Moreover, TUNEL staining showed that the apoptosis level was significantly higher in the pikeperch (*Sander lucioperca*) when the water temperature increased from 30 to 34 °C [15]. Therefore, there is a need to improve the heat stress resistance of aquatic animals through nutritional manipulations.

Methionine is a major limiting amino acid and an essential nutrient in aquatic animals [16]. Methionine can promote lipid mobilization to provide energy for growth and protein deposition by regulating nutrients metabolism in aquatic animals [17,18]. In addition, methionine enhances antioxidant capacity and immunity in aquatic animals [17,19]. Methionine residues can be used as endogenous antioxidants to alleviate injury caused by excess ROS [20,21]. Moreover, dietary methionine could modulate the immunity of grass carp *Ctenopharyngodon idella* through the Nrf2 and MAPK pathways [19]. Similarly, dietary methionine could upregulate the mRNA expression of heat shock protein 70 in the gut to protect intestinal mucosa damage in broiler chickens from heat stress [22]. Therefore, dietary methionine may alleviate the adverse reactions caused by high-water-temperature stress and ensure the normal metabolism of protein.

*E. sinensis* is an economic crab due to its high nutritional value and taste and is mostly cultivated in the subtropical zone [23]. The optimal water temperature for *E. sinensis* is 19–25 °C [23]. However, the water temperature in summer usually ranges from 28 to 32 °C, which exceeds the optimum water temperature for *E. sinensis* and induces heat stress [23]. Crabs are crustaceans with a weak thermoregulation ability, and several studies have reported that heat stress could adversely affect growth, antioxidant ability, immunity reproduction and aquaculture benefits [24,25,26,27,28]. Therefore, this study aimed to investigate the adverse effects of chronic heat stress and the mitigative effect of dietary methionine on juvenile *E. sinensis*. This may provide a theoretical basis for developing methionine as a feed additive for juvenile *E. sinensis*, and a new approach was proposed to reduce chronic heat stress in crabs.

## 2. Materials and Methods

### 2.1. Experimental Diets

The formulation and proximate chemical composition of the experimental diets are shown in Table 1. Three experimental diets were formulated to contain three methionine levels (0.49%, 1.29% and 2.09% of the diet). Coated methionine was used in experimental diets, and the purity of the methionine was 50% of the compound. The amino acid concentrations measured in the diets are shown in Table 2. The protein sources in the diets were mainly fermented soybean meal, cottonseed meal, corn gluten meal and chicken meal, and the lipid sources were mainly fish oil, cholesterol and lecithin. All ingredients were crushed using a grinder, sieved, and mixed well. Subsequently, oil and deionized water (containing choline chloride) were added. A double-screw-press pelletizer was used to produce pellets with a 2.5 mm diameter. The diets were dried until the moisture content was <10%. The diets were sealed in vacuum bags and stored at −20 °C until use.

### 2.2. Design, Sampling and Growth Measurement

The trial was carried out in the laboratory in Huzhou, Zhejiang. The crabs used in this study were purchased from a local farm in Nantong, Jiangsu. All crabs were distributed into 300 L tanks (100 × 80 × 60 cm) and fed a commercial diet for one week before the trial. Subsequently, 1200 juvenile crabs (initial average weight 0.71 ± 0.01 g) were randomly allocated. The male and female crab ratio was one-to-one. Five tanks were set at normal water temperature (water temperature = 24 °C), while the other five were set at high water temperature (water temperature = 30 °C). Six treatment combinations were used in this study. The normal-water-temperature treatment contained (1) low dietary methionine (NT-LM), (2) medium dietary methionine (NT-MM), and (3) high dietary methionine (NT-HM). The high-water-temperature treatment contained (4) low dietary methionine level diets (HT-LM), (5) medium dietary methionine (HT-MM), and (6) high dietary methionine (HT-HM). The high-water-temperature reached 30 °C at 2 °C per day. The water temperature was checked every 8 h using a digital thermometer. Five groups of plastic corrugated pipes, tiles and other shelters were placed at the bottom of each tank to reduce the occurrence of cannibalism among individuals. Crabs were fed the corresponding diets at 4% of their body weight at 14:20 h and 20:30 h twice daily during the feeding trial. The feed intake (FI) of crabs was recorded daily. After feeding, all tanks were cleaned daily by siphoning out the feed residue and faeces, and the water of 30% tank volume was exchanged daily to maintain water quality. The dissolved oxygen was always >7.0 mg/L, and the pH ranged from 7.5 to 8.5. Total ammonia nitrogen was below 0.05 mg/L. Crabs were reared under the natural photoperiod (12 h light and 12 h dark).

At the end of the 6-week feeding trial, crabs fasted for 24 h before sampling and then the number and weight of the remaining crabs in each tank were recorded. Five crabs were randomly selected from each tank and stored at −20 °C for subsequent whole-body proximate analysis. Another 10 crabs from each tank were placed on crushed ice for anesthesia, and then we extracted hemolymph from the base of the leg joints with a 1 mL syringe and collected it in a 1.5 mL centrifuge tube. The haemolymph was stored at 4 °C overnight. The haemolymph in the centrifuge tube was thoroughly crushed with a syringe needle until there was no obvious clot and then centrifuged at 7000 rpm for 10 min. Then, the supernatant was separated and stored at −80 °C. The protocols for animal use complied with the guidance of the Care and Use of Laboratory Animals in China (20201001).

The WG, specific growth rate (SGR), survival, MR, FI, feed conversion ratio (FCR) and protein efficiency ratio (PER) were calculated according to the following formulas:

WG (%) = (Wt − W0)/W0 × 100(1)

SGR (% day^−1^) = [lnWt − lnW0]/T × 100 (2)

Survival (%) = Nf/Ni × 100(3)

MR = 100 × Nm/Nf(4)

FI = Total feed weight/final number(5)

FCR = Fi/(Wt − W0 + Wd)(6)

PER = (Wt − W0)/Fi × Pi(7)

where Wt, W0 and Wd were the means of final wet body weight, initial wet body weight and dead crab weight, respectively. Nf, Ni, and Nm, were the final crab numbers, initial crab numbers and molting numbers, respectively. T and Fi were the duration (days) of the experiment and food intake, and Pi was the feed protein content (dry weight).

### 2.3. Chemical Composition Analysis

The contents of moisture, ash, crude lipid and crude protein in the experimental diets and whole-body were determined by the standard method [29]. Four duplicates were used in each measured parameter (*n* = 4). We dried the sample at 105 °C to constant weight to determine moisture content. The crude protein content was quantified by digestion of the samples with concentrated sulfuric acid, and then by Kjeldahl TM 8200 (Foss, Hoganas, Sweden). In addition, we determined the total lipid content [29]. First, we used the chloroform–methanol solution and 0.37 mol/L KCL solution for extraction, and finally used a vacuum-drying oven (Jinghong Experimental Equipment, Shanghai, China) for quantification. To determine the ash content, we first placed the sample in an electric furnace for complete carbonization and then placed the sample in a muffle furnace (PCD-E3000, Shanghai, China).

### 2.4. Enzyme Activity Assay

The hepatopancreas homogenate supernatant was used to determine enzyme activities in the hepatopancreas using a diagnostic kit (Nanjing Jiancheng Institute of Bioengineering, Nanjing, China). The triglycerides (TG) content is directly proportional to the color of quinone compounds. We determined the absorbance value to calculate TG’s content. The reaction system of xanthine and xanthine oxidase produces superoxide anion (O^2−^). The O^2−^ can reduce nitrogen blue tetrazolium to produce blue methamphetamine to determine the activity of superoxide dismutase (SOD). Malondialdehyde (MDA) can be condensed with thiobarbituric acid to form a red product with a maximum absorption peak at 532 nm. Reduced glutathione (GSH) reacts with 5,5′-dithiobis-bis-(2-nitrobenoic acid) (DTNB) to produce yellow 2-nitro-5 mercaptobenoic acid, which has a characteristic absorption peak at 412 nm. GSH peroxidase (GSH-Px) reacts H_2_O_2_ with reduced GSH to form H_2_O and oxidized GSH. The decomposition of H_2_O_2_ by catalase (CAT) stopped when ammonium molybdate was added, and the remaining H_2_O_2_ reacted with ammonium molybdate to form a pale-yellow complex. Six samples per group were used in the test.

### 2.5. Analysis of Gene Expression

Total RNA was extracted from the hepatopancreas by RNAiso Plus (Takara, Dalian, China) according to the manufacturer’s instructions. The RNA concentration and quality in each sample were determined using a Nano Drop 2000 spectrophotometer (Thermo, MA, USA) and 1% agarose gel. When the optimal density of A260/A280 was between 1.9 and 2.0, genomic DNA was removed by reaction with 1 μg total RNA at 42 °C for 2 min, followed by Hi-Script II Q RT SuperMix for qPCR (+gDNA wiper) (Vazyme, Nanjing, China) obtained complementary DNA (cDNA) samples according to the instructions. Real-time PCR reactions were determined using a QuantStudio 6 Flex Real-time PCR system (Thermo Fisher Scientific). The real-time PCR reaction system was 20 μL: 0.4 μL of each forward and reverse primer (concentration of primers was 10 mM), 2 μL of 5-fold-diluted cDNA template, 7.2 μL of nuclease-free water, and 10 μL of 2× ChamQ Universal SYBR qPCR Master Mix (Vazyme, Nanjing, China). Real-time PCR reaction amplification proceeded according to Han et al. (2019). After the amplification program, the melting curve program was further run (from 60 °C to 95 °C at a rate of 0.1 °C/s) to test the specificity of the amplification reaction. The β-actin was used as a reference gene [30]. The relative mRNA level of the target gene was selected as the calibrator for crabs fed low-dietary-methionine diets at 24 °C, and the relative expression of the target gene was determined by the formula 2^−ΔΔCT^ [31]. The target genes that were used are shown in Table 3. Eight samples per group were used in the test.

### 2.6. Statistical Analysis

SPSS 23.0 for Windows (SPSS, Michigan Avenue, Chicago, IL, USA) was used for statistical analysis. Levene’s equal variance test and the Shapiro–Wilk test were used to check the homogeneity of variance and normal distribution of all data. Two-way analysis ANOVA was used to determine whether there was an interaction between dietary methionine levels and water temperature. Under the same water temperature conditions, one-way analysis of variance (ANOVA) was used to analyze whether there was a significant difference between crabs at different methionine levels. If one-way ANOVA was significant, Duncan’s range test further analyzed the significance of different treatments. The *t*-test determined the significant differences between crabs at the control and high temperatures at the same dietary methionine level. Significance was set at *p* < 0.05. The data are represented as the mean ± standard error of the mean (SEM).

## 3. Results

### 3.1. Growth Performance

In the NT groups, crabs in the NT-MM group had higher WG, SGR, survival, MR and PER than crabs in the NT-LM groups (*p* < 0.05) (Table 4). Crabs had lower WG, SGR, MR and PER in the NT group than in the HT group (Table 4). There was no significant difference in FI between groups at 24 °C. The FI significantly increased under chronic heat stress regardless of dietary methionine levels. Crabs in the NT-MM group had the lowest FCR among all NT groups. Chronic heat stress significantly decreased the FCR and survival when crabs were fed diets with low or medium methionine levels. However, survival was higher in the HT-HM groups than in the HT-LM and HT-MM groups (*p* < 0.05). There was no significant difference in survival and FCR in the NT-HM and HT-HM groups.

### 3.2. Whole-Body Proximate Composition

There was no significant difference in the whole-body moisture and ash content of crabs among the groups (Table 5). At 24 °C, whole-body crude protein increased as the amount of dietary methionine added to the diet increased. Crabs in the HT-MM group had lower whole-body crude protein than crabs in the NT-MM group (*p* < 0.05). Meanwhile, the whole-body crude protein was significantly reduced in the HT-LM and HT-MM groups compared with the HT-HM groups. Crabs in the NT-MM and NT-HM groups had higher whole-body crude lipid content than crabs in the NT-LM groups. The whole-body crude lipid content was higher in the HT-LM than in the NT-LM (*p* < 0.05). In addition, crabs in the HT-MM group showed higher whole-body crude lipid content than those in the NT-MM group (*p* < 0.05). In the high-dietary-methionine level diets, there was no significant difference in the whole-body crude lipid between crabs cultivated at normal and high water temperatures.

### 3.3. Hepatopancreas Lipid, Triglyceride (TG) and Gene Expressions of Lipid Metabolism

**Figure 1:**
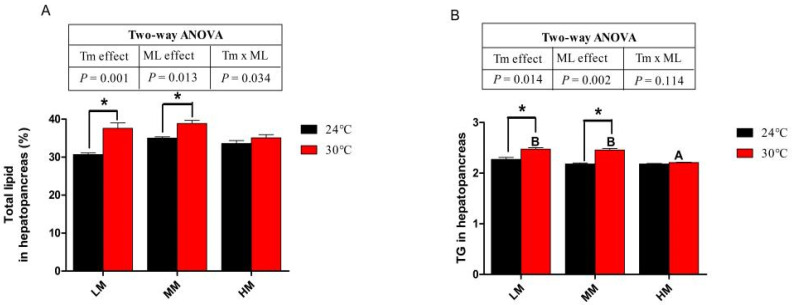
The total lipid (**A**) and triglyceride contents (**B**) in the hepatopancreas of juvenile *E. sinensis* fed the experimental diets at normal (24 °C)/high water temperature (30 °C). * means that there is a significant difference between different temperatures at the same dietary methionine level (*p* < 0.05). The A and B indicate significant differences among crabs fed diets with different dietary methionine levels at high water temperature (*p* < 0.05).

**Figure 2:**
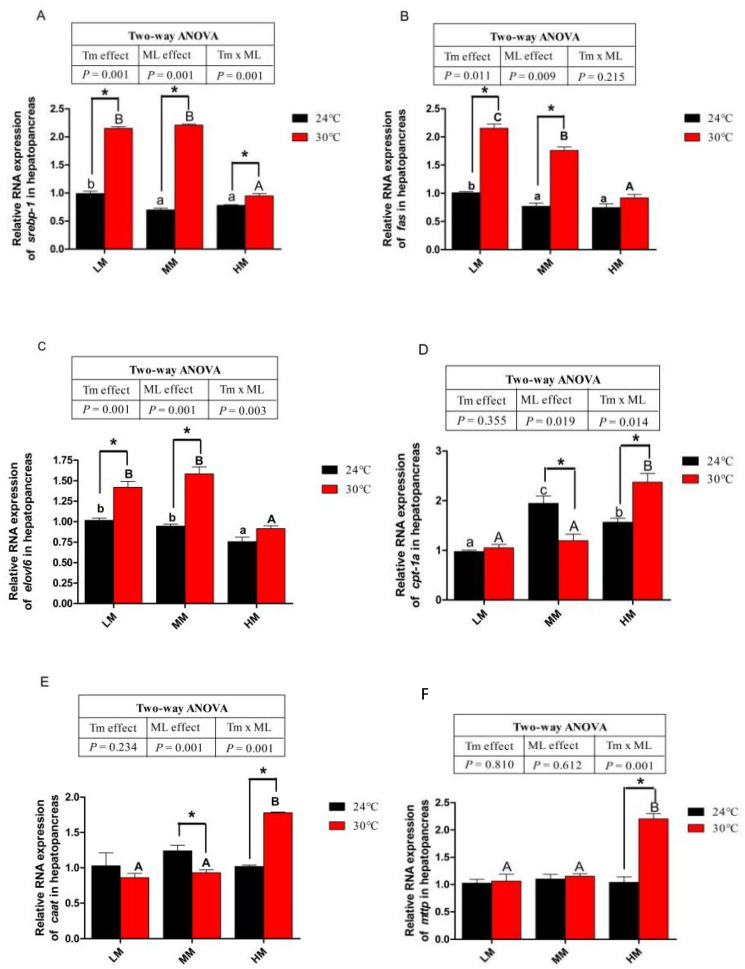
The lipid metabolism of juvenile *E. sinensis* fed the experimental diets at normal water temperature (24 °C)/high water temperature (30 °C). (**A**) *srebp-1*: sterol-regulatory element binding protein 1; (**B**) *fas*: fatty acid synthase; (**C**) *elovl6*: elongase of very-long-chain fatty acids 6; (**D**) *cpt-1α*: carnitine palmitoyl transterase 1*α*; (**E**) *caat*: carnitine acetyltransferase; (**F**) *mttp*: microsomal triglyceride transfer protein. * means that there is a significant difference between different temperatures at the same dietary methionine level (*p* < 0.05). The a, b and c indicate significant differences among crabs fed diets with different dietary methionine levels at normal temperature (*p* < 0.05). The A and B indicate significant differences among crabs fed diets with different dietary methionine levels at high water temperature (*p* < 0.05).

The total lipid and TG content in the hepatopancreas of crabs were shown in Figure 1. There was no significant difference in total lipid and TG content in the hepatopancreas between NT groups (Figure 1A,B). Chronic heat stress significantly increased total lipid and TG when crabs were fed diets with low and medium methionine levels (Figure 1A,B). There was no difference in total lipid or TG content in the hepatopancreas between the NT-HM and HT-HM groups (*p* > 0.05). Crabs in the NT-MM and NT-HM groups showed lower gene expression levels of sterol-regulatory element binding protein 1 (*srebp-1*), fatty acid synthase (*fas*) than crabs in the NT-LM group (*p* < 0.05, Figure 2A,B). The gene expression levels of elongase of very-long-chain fatty acid 6 (*elovl6*) in the NT-HM group was significantly lower than in the NT-LM and NT-MM groups (Figure 2C). The gene expressions of *srebp-1*, *fas* and *elovl6* in the HT-LM group were higher than those in the NT-LM group (*p* < 0.05, Figure 2A–C). Furthermore, the mRNA abundances of these genes in the HT-MM group were also higher than those in the NT-MM group. However, the mRNA levels of these genes in the HT-HM group were lower than those in the HT-LM and HM-MM groups (*p* < 0.05). There was no significant difference in *fas* and *elovl6* gene expression between the NT-HM and HT-HM groups. Crabs in the NT-MM group showed significantly higher gene expression of carnitine palmitoyl transterase 1*α* (*cpt-1α*) than crabs in the NT-LM and NT-HM groups, and there was no significant difference in the gene expression of carnitine acetyltransferase (*caat*) and microsomal triglyceride transfer protein (*mttp*) among crab in the NT groups (Figure 2D–F). The gene expression of *cpt-1α* and *caat* in the NT-MM was higher than those in the HT-MM (*p* < 0.05, Figure 2D,E). In addition, high-dietary-methionine supplementation reversed the gene expression decline in *cpt-1a*, *caat* and *mttp* in the hepatopancreas induced by chronic heat stress (*p* < 0.05, Figure 2D–F). The gene expression of cpt-1a, *caat* and *mttp* in the HT-HM group was higher than that in the NT-HM group (*p* < 0.05).

### 3.4. Hepatopancreas and Muscle Crude Protein Content

**Figure 3:**
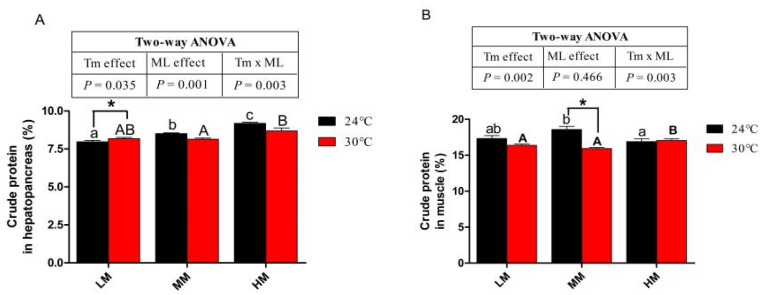
The crude protein content in hepatopancreas (**A**) and the crude protein in muscle (**B**) of juvenile *E. sinensis* fed the experimental diets at normal (24 °C)/high water temperature (30 °C). * means that there is a significant difference between different temperatures at the same dietary methionine level (*p* < 0.05). The a, b and c indicate significant differences among crabs fed diets with different dietary methionine levels at normal temperature (*p* < 0.05). The A and B indicate significant differences among crabs fed diets with different dietary methionine levels at high water temperature (*p* < 0.05).

At 24 °C, with the increase in dietary methionine content in the diet, the crude protein content of hepatopancreas also increased (Figure 3A). The crude protein content of hepatopancreas in the HT-MM group was lower than that in the NT-MM group. Furthermore, crabs in the HT-HM group also presented reduced crude protein content of hepatopancreas compared with those in the NT-HM group. The crabs in the HT-HM group had a higher crude protein content in the hepatopancreas than crabs in the HT-LM and HT-MM groups (*p* > 0.05, Figure 3A). Crabs in the NT-MM group showed the highest crude protein content of muscle among crabs in the NT groups (Figure 3B). At low dietary methionine levels, crabs in the HT group had lower muscle protein than those in the NT group (Figure 3B). Similarly, the crabs in the HT-MM groups showed lower muscle protein than crabs in the NT-MM groups (*p* < 0.05). However, the HT-HM group presented a higher crude protein content of muscle than the HT-LM and HT-MM groups (*p* < 0.05). At high dietary methionine levels, there was no significant difference in muscle protein between crabs cultivated at 24 °C and 30 °C.

### 3.5. Antioxidative Capacity

**Figure 4:**
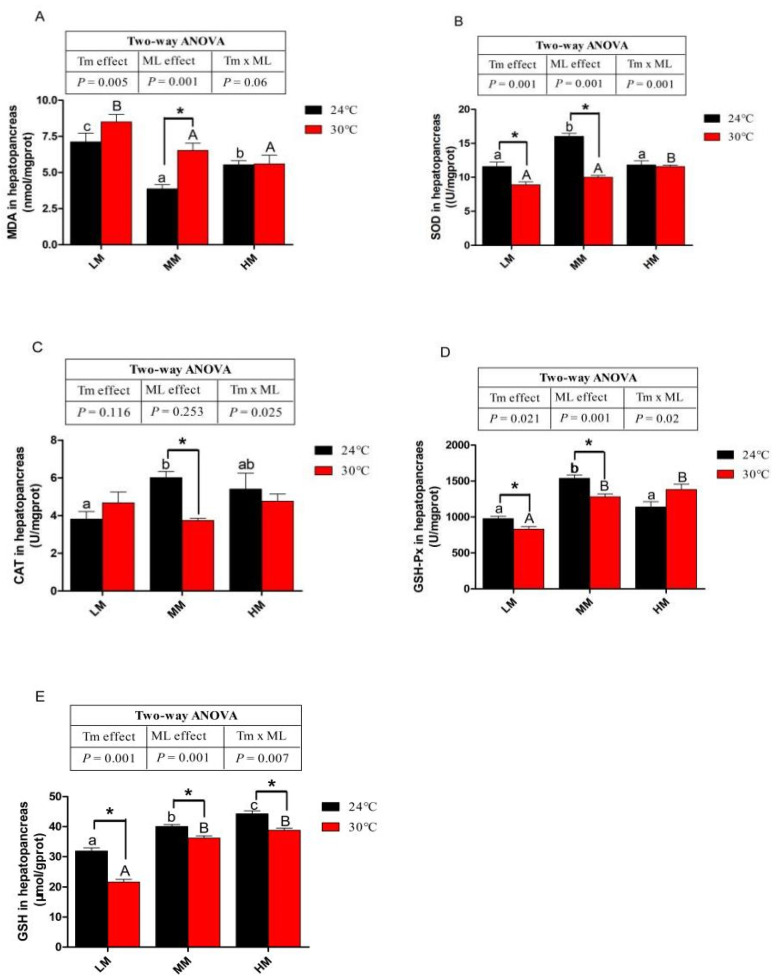
The antioxidative capacity of juvenile *E. sinensis* fed the experimental diets at normal (24 °C)/high water temperature (30 °C). (**A**) MDA: malondialdehyde; (**B**) SOD: superoxide dismutase; (**C**) CAT: catalase; (**D**) GSH-Px: glutathione peroxidase; (**E**) GSH content. * means that there is a significant difference between different temperatures at the same dietary methionine level (*p* < 0.05). The a, b and c indicate significant differences among crabs fed diets with different dietary methionine levels at normal temperature (*p* < 0.05). The A and B indicate significant differences among crabs fed diets with different dietary methionine levels at high water temperature (*p* < 0.05).

Crabs in the NT-MM groups had lower MDA content in the hepatopancreas than crabs in the NT-LM and NT-HM groups (*p* < 0.05, Figure 4A). Chronic heat stress increased the MDA content in the crab hepatopancreas at low and medium dietary methionine levels. However, high dietary methionine decreased the MDA content in the hepatopancreas compared to those fed low-dietary-methionine diets under chronic heat stress (*p* < 0.05, Figure 4A). Crabs in the NT-MM groups had higher SOD, CAT and GSH-Px activities than crabs in the NT-LM and NT-MM groups (Figure 4B–D). At 24 °C, with the increase in dietary methionine content in the diet, the GSH content in the hepatopancreas also increased (*p* < 0.05, Figure 4E). At low and medium dietary methionine levels, crabs cultivated at a high water temperature had lower SOD and GSH-Px activities and GSH content than those cultivated at normal water temperature (*p* < 0.05, Figure 4B,D,E). The crabs fed high-dietary-methionine level diets increased SOD activity compared with those fed low and medium diets under chronic heat stress (*p* < 0.05, Figure 4B). Crabs in the HT-MM and HT-HM groups showed higher GSH-Px activity and GSH content than crabs in the HT-LM group (*p* < 0.05, Figure 4D,E). There was no significant difference in CAT activity between HT groups (Figure 4C). The CAT activities were reduced in the HT-MM groups compared with those in the NT-MM groups (*p* < 0.05, Figure 4C). No significant difference was found in the antioxidant enzyme activities of the crabs between the NT-HM and HT-HM groups.

### 3.6. Expression of Genes about Apoptotic Factors and Tlrs Pathway

**Figure 5:**
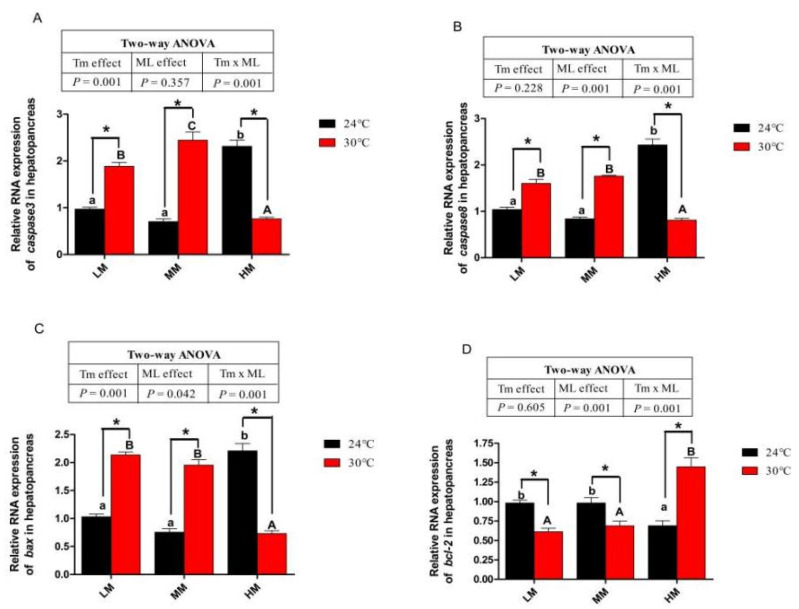
The apoptosis of juvenile *E. sinensis* fed the experimental diets at normal water temperature (24 °C)/high water temperature (30 °C). (**A**) *Caspase 3*: cysteine–aspartic acid protease 3; (**B**) *caspase 8*: cysteine–aspartic acid protease 8; (**C**) *bax*: B-cell lymphoma-2-associated X; (**D**) *bcl-2*: B-cell lymphoma-2. * means that there is a significant difference between different temperatures at the same dietary methionine level (*p* < 0.05). The a and b indicate significant differences among crabs fed diets with different dietary methionine levels at normal temperature (*p* < 0.05). The A, B and C indicate significant differences among crabs fed diets with different dietary methionine levels at high water temperature (*p* < 0.05).

**Figure 6:**
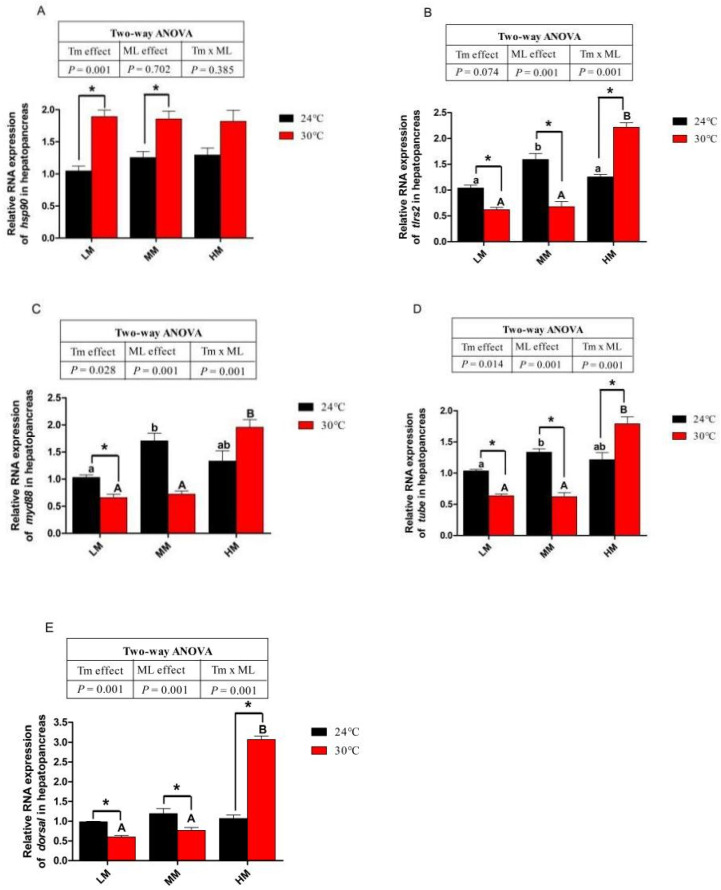
The toll-like receptor-related pathway of juvenile *E. sinensis* fed the experimental diets at normal water temperature (24 °C)/high water temperature (30 °C). (**A**) *Hsp90*: heat shock protein 90; (**B**) *tlr2*: toll-like receptor 2; (**C**) *myd88*: myeloid differentiation factor 88; (**D**) *tube*; (**E**) *dorsal*. * means that there is a significant difference between different temperatures at the same dietary methionine level (*p* < 0.05). The a and b indicate significant differences among crabs fed diets with different dietary methionine levels at normal temperature (*p* < 0.05). The A and B indicate significant differences among crabs fed diets with different dietary methionine levels at high water temperature (*p* < 0.05).

Crabs in the NT-HM group showed higher gene expression of cysteine-aspartic acid protease 3 (*caspase3*), cysteine-aspartic acid protease 8 (*caspase8*) and B-cell lymphoma-2-associated X (*bax*) than those in the NT-LM and HT-MM groups (*p* < 0.05, Figure 5A–C). Crabs in the NT-HM group showed a lower gene expression of B-cell lymphoma-2 (*bcl-2*) than those in the NT-LM and NT-MM groups (*p* < 0.05, Figure 5D). As shown in Figure 5A–D, crabs in the HT-LM group presented a significantly lower gene expression of *bcl-2* and higher gene expression of *caspase3*, *caspase8* and *bax* than those in the NT-LM group. The gene expressions of *caspase3*, *caspase8* and *bax* significantly increased, and the gene expression of *bcl-2* was significantly reduced in the HT-MM group compared with the NT-MM group (Figure 5A–D). The mRNA abundance of *caspase 3*, *caspase 8* and *bax* in the HT-HM group was lower than that in the HT-LM and HT-MM groups (*p* < 0.05). In addition, crabs in the HT-HM groups had lower *caspase3*, *caspase8* and *bax* and higher *bcl-2* gene expression than crabs in the NT-HM groups (*p* < 0.05). There was no significant difference in *hsp90* gene expression among NT groups (Figure 6A). Chronic heat stress significantly increased *hsp90* gene expression in crabs, regardless of dietary methionine levels (Figure 6A). There was no difference in *hsp90* mRNA level among the HT groups (*p* > 0.05). Crabs in the NT-MM groups showed highest gene expression levels of toll-like receptor 2 (*tlrs2*), myeloid differentiation factor 88 (*myd88*), and *tube* among crabs in the NT groups (Figure 6B–D). There was no significant difference in the gene expression level of *dorsal* among crabs in the NT groups (Figure 6E). The gene expression levels of *tlrs2*, *myd88*, *tube* and *dorsal* in the HT-LM and HT-MM groups were significantly lower than those in the NT-LM and NT-MM groups, respectively (Figure 6B–E). However, high-dietary-methionine supplementation increased the mRNA abundance of these genes under chronic heat stress (*p* < 0.05, Figure 6B–E). The crabs in the HT-HM group showed higher gene expression levels of *tlrs2*, *myd88*, *tube* and *dorsal* than those in the HT-LM and HT-MM groups (*p* < 0.05, Figure 6B–E). Furthermore, the gene expressions of *tlrs2*, *myd88*, *tube* and *dorsal* in the HT-HM groups were higher than those in the NT-HM groups (*p* < 0.05).

## 4. Discussion

In this study, the molting frequency rate and weight gain of juvenile crabs exposed to heat stress significantly increased. Previous studies on *A. minor* and *S. salar* reported that heat stress could inhibit growth [7,8]. This may be because a higher metabolic rate and nutrient consumption occur under heat stress, resulting in an insufficient energy supply for growth [12]. However, in the current study, high water temperature significantly promoted the growth of juvenile *E. sinensis*, indicating species differences. The weight gain of the 0.9 g crabs significantly increased at 28 °C, and similar results were also observed in Pacific white shrimp (*Litopenaeus vannamei*) [10,32,33]. Meanwhile, the present study found that the feed conversion ratio significantly decreased and feed intake significantly increased after heat stress, indicating that *E. sinensis* ate more under heat stress, and a high water temperature can promote nutrient accumulation to benefit the growth and development of crabs. As aquatic animals can use more energy from feed for growth under heat stress, a significant increase in feed intake might reduce nutrient consumption and promote nutrient accumulation (such as lipid) [34]. This may be a reason for the significant improvement in growth performance in juvenile *E. sinensis* [34].

In the present study, a high water temperature significantly decreased survival, which is consistent with the high mortality of red claw crayfish (*Cherax quadricarinatus*) exposed to 38 °C [35]. Although chronic heat stress promoted growth, survival significantly decreased, indicating that the increased weight gain caused by a high water temperature might be abnormal and harmful to *E. sinensis* [36]. In contrast, the addition of high dietary methionine to the diets improved the survival of juvenile *E. sinensis* by heat stress. Although heat stress might result in oxidative stress, tissue damage and nutrient consumption in the hepatopancreas, these adverse effects reduce the survival of *E. sinensis* [36]. Studies have shown that dietary methionine can improve the survival rate of *E. sinensis,* and methionine can enhance the anti-stress ability by improving the antioxidant capacity of aquatic animals [37,38]. Moreover, methionine can promote nutrient accumulation in the body and reshape the energy metabolism to convert more nutrients into energy and resist stress in Nile tilapia (*Oreochromis niloticus*) [39]. As a result, chronic heat stress affected the feeding, molting frequency rate, and weight gain and significantly decreased the survival of juvenile *E. sinensis*. However, a high level of dietary methionine could counteract the adverse effect of heat stress on survival.

Subsequently, the present study found that a high water temperature led to the lipid deposition of juvenile *E. sinensis*, especially in the hepatopancreas. Similarly, continuous heat stress results in lipid accumulation in the liver of rainbow trout (*Oncorhynchus mykiss*) [40]. Heat stress could increase lipid synthesis by upregulating the activity of s*rebp-1*, which might be one of the mechanisms leading to lipid accumulation in the liver [41]. As a membrane-bound transcription factor, *srebp-1* participates in lipid metabolism by activating genes related to fatty acid synthesis, such as *fas* and *elovl6* [41,42]. In this study, genes related to lipid synthesis were upregulated in the hepatopancreas at high water temperatures. Heat stress also significantly increased fatty acid synthase activities, mRNA expression of *srebp-1* and lipid content in the liver of broiler chickens [41]. In addition, *cpt-1α* and *caat* are fatty acid β-oxidation-related genes, and *mttp* is a gene associated with very-low-density lipoprotein assembly to promote lipid transport outside the liver [43,44]. In this study, the expression of these genes was downregulated under chronic heat stress. These results indicated that the lipid deposition in the hepatopancreas might be due to the impairment of hepatopancreas function under heat stress, resulting in lipid absorption, metabolism and transport disorders [40]. However, adding high dietary methionine changed the occurrence of the above phenomenon and alleviated the abnormal accumulation of lipid. A previous study also revealed that a high level of dietary methionine could upregulate the genes expression of fatty acid β-oxidation and relieve lipid accumulation in the liver in large yellow croaker (*Larimichthys crocea*) fed high-lipid diets [45]. In this study, dietary methionine increased the total whole-body lipid at normal water temperature. Previous studies have found that dietary methionine supplementation can increase the content of phospholipids, so the increased content of total lipid might be due to the synthesis of phospholipids, which could reduce the abnormal lipid accumulation caused by chronic heat stress [45,46]. Another possible reason for this is that dietary methionine affects the synthesis of lipoproteins [46]. Lipoprotein could promote the transport of hepatic TG out of the liver in the form of VLDL to reduce lipid deposition in the liver [46]. In summary, dietary supplementation with a high level of methionine could effectively alleviate metabolic lipid disorders caused by chronic heat stress in juvenile *E. sinensis*.

The current study also found that the crude protein content of the body, hepatopancreas and muscle of juvenile *E. sinensis* declined under chronic heat stress. Pyruvate kinase (PK) plays a vital role in glycolysis and carbon flows, but high water temperature reduces the activity of PK, thus affecting the utilization of carbohydrates [40]. In addition, the lipid utilization of juvenile *E. sinensis* was blocked under chronic stress [40]. Therefore, protein becomes the main nutrient in the energy of aquatic animals under high water temperatures [6,47]. The decrease in the protein content of juvenile *E. sinensis* caused by heat stress might be related to the use of protein as a substitute for carbohydrates and lipidFlipid for energy supply [48]. Similarly, heat stress caused the breakdown of breast muscle proteins in broilers, providing amino acid substrates for energy supply [49]. In the current study, the addition of high-level dietary methionine increased the protein deposition of juvenile *E. sinensis* under chronic heat stress. Dietary methionine also can increase the muscle protein content of juvenile yellow river carp (*Cyprinus carpio hematopterus*) [50]. Methionine can activate the activity of insulin-like growth factor-1, which triggers the activation of phosphatidylinositol 3-kinase/protein kinase B/mammalian target of the rapamycin pathway to promote protein deposition [51]. Overall, the high level of dietary methionine could mitigate protein degradation in *E. sinensis* under chronic heat stress.

With the lipid deposition and ROS accumulation induced by heat stress, lipid peroxidation easily occurs in the polyunsaturated fatty acids and fatty acids in the phospholipids of biofilms [52]. As a final product of lipid peroxidation, MDA content is an important parameter reflecting the degree of peroxidation damage and oxidative stress [52]. The SOD, CAT and GSH-Px are essential indicators of the antioxidant defense system in animals and plants and can effectively eliminate ROS in the body, reduce lipid peroxidation reactions and protect against oxidative stress [53]. The GSH is the essential substrate for GSH-Px to decompose peroxides [54]. In the current study, the MDA content in the hepatopancreas increased, and the antioxidant enzyme activities were downregulated when the water temperature was 30 °C. This may be because more peroxidation occurred in fatty acids, caused by the increased ROS accumulation and lipid deposition under heat stress in juvenile *E. sinensis*. However, this study suggests that the high-dietary-methionine level diets effectively increased the antioxidant capacity and decreased the MDA content under chronic heat stress. The present study also found that the addition of a medium level of dietary methionine in the diets improved the antioxidant capacity of crabs at 24 °C, while the addition of a high level of dietary methionine in the diets might lead to metabolic methionine disorders and reduce the antioxidant capacity. Similarly, dietary methionine can improve the antioxidant enzyme activities of gilthead sea bream (*Sparus aurata*) under acute hypoxic conditions [55]. Methionine is a precursor of GSH, which can protect cells from oxidative stress by reducing ROS [56]. Moreover, cysteine synthesized by methionine through the trans-sulfur pathway is involved in the synthesis of some antioxidants, such as taurine, in vivo [57]. These metabolites probably improved the antioxidant capacity and reduced lipid peroxidation of juvenile *E. sinensis* under heat stress [57]. Collectively, high-dietary-methionine level diets could effectively improve antioxidant capacity to alleviate the oxidative stress caused by chronic heat stress.

Oxidative stress can also induce and trigger cell apoptosis [58]. Caspase is a cysteine–aspartate-specific protease in the cytoplasm and plays a crucial role in cell apoptosis [58]. The *caspase8* is upstream of the apoptotic pathway, and *caspase3* is downstream of the cascade [59]. Moreover, the high *bax* expression could promote apoptosis [60]. Conversely, *bcl-2* protein inhibits apoptosis by protecting the mitochondrial membrane and reducing cytochrome C release into the cytoplasm [61]. The present study found that dietary supplementation of 1.29% methionine did not activate the anti-apoptosis ability under normal water temperatures, while the actual measured value of the medium dietary supplemented level of methionine in the present study was 1.05%. The previous study found that the dietary supplementation of 1.06% methionine enhanced the anti-apoptosis ability of crabs [37]. This might be due to the different formulas used in these two studies. The formula in this study had no fish meal supplementation, while the dietary formula with 15% fish meal content was used in the previous study [37]. Moreover, in the present study, the absence of fishmeal in diets might cause taurine deficiencies and reduce the anti-apoptosis capacity of crabs [57]. The present study also observed that insufficient dietary methionine increased the transcription level of apoptosis-related genes exposed to chronic heat stress, whereas high dietary methionine levels could reverse this trend. The results showed that heat stress might trigger apoptosis by inducing oxidative stress, and high dietary methionine levels could effectively alleviate this toxic response. In addition, compared with normal water temperature, this study found that crabs may need more dietary methionine to resist the adverse effects of oxidative stress under chronic stress. Therefore, dietary methionine may play a different role in crab under normal or high temperatures. The protective effect of methionine may be achieved by activating the Nrf2/bcl-2 pathway to prevent cells from free radical attack [62].

When cells are subjected to stress or damage, especially heat stress, heat shock proteins (HSPs) play a crucial role as highly conserved proteins [63]. Increased HSPs gene expression is used as a marker of heat stress [64]. The Hsp90 is the most widespread member of the HSP family, which maintains cell structural integrity, refolds abnormally folded proteins and improves the heat resistance of cells [65,66,67,68,69,70]. In this study, the expression of *hsp90* mRNA was significantly upregulated in juvenile crabs exposed to 30 °C compared with the 24 °C group, suggesting that the response mechanism can be activated by 30 °C. Similarly, heat stress also increased the mRNA expression of HSPs in *O. niloticus* [71]. In addition, heat stress could activate downstream signals (*Tlrs* pathway) to constitute a unique natural immune mode, alleviating the oxidative stress caused by heat stress and maintaining the balance of the body’s internal environment [72]. The *Tlrs* pathway relies on *myd88* to transmit signals to downstream dorsal molecules, producing an inflammatory response, completing the immune response, and protecting the body [73]. Tube is the connector between *myd88* and pelle and plays a vital role in the immune response [73]. In the current study, juvenile crabs under chronic heat stress had a lower mRNA abundance of *Tlrs*-related genes, blocking the activation of the *Tlrs*-pathway and failing to generate an immune response, making crabs unable to resist heat stress [74]. However, high-dietary-methionine level diets could reverse the repressed expression of *Tlrs*-related genes exposed to chronic heat stress. Dietary methionine increased the white blood cell mass and activated the *Tlrs*-pathway to resist abdominal inflammation of *European seabass* (*Dicentrarchus labrax*) subjected to UV-killed *Photobacterium damselae* subsp. *Piscicida* [75]. Methionine is a significant methyl donor, and the higher the methyl input, the more leukocytes there are, upregulating the *Tlrs*-pathway to improve the ability to resist heat stress [76]. This study shows that a high-dietary-methionine level diet could effectively alleviate heat-induced oxidative stress damage. Furthermore, dietary methionine can enhance the immune function of juvenile crabs by activating the *Tlrs*-pathway to generate the immune response and improve the health *E. sinensis* under chronic heat stress.

## 5. Conclusions

The FI and growth performance increased under chronic heat stress. However, chronic heat stress also induced low survival, lipid accumulation, protein degradation, oxidative stress, immunosuppression and apoptosis. Importantly, when a high dietary methionine level was added to the diet, the WG and survival were improved simultaneously, and lipid accumulation and protein reduction induced by chronic heat stress were mitigated. High dietary methionine supplementation also reduced lipid peroxidation under chronic heat stress by improving antioxidant capacity. In addition, oxidative stress was alleviated by inhibiting apoptosis and mobilizing *Tlrs*-related pathways when crabs were fed high dietary methionine to improve crab health under chronic heat stress. This study sheds light on the application of methionine in the diet of aquatic invertebrates during chronic heat stress.

## Figures and Tables

**Table 1:** Formulation and chemical proximate composition of experimental diets (dry matter basis, %).

Ingredients	Experiment Diets		
	LM	MM	HM
Fermented soybean meal	30	30	30
Cottonseed meal	15	15	15
Corn gluten meal	12	12	12
Chicken meal	12	12	12
α-starch	12	12	12
Fish oil	4.5	4.5	4.5
Lecithin	1	1	1
Cholesterol	0.5	0.5	0.5
Choline chloride	0.5	0.5	0.5
Vitamin premix ^a^	3	3	3
Mineral premix ^b^	2	2	2
Coated lysine	1.37	1.37	1.37
Butylated hydroxytoluene	0.05	0.05	0.05
Cellulose	2.48	1.68	0.88
Carboxymethyl cellulose	2	2	2
Glycine	1.6	0.8	0
Coated methionine	0	1.6	3.2
Proximate composition			
Moisture	13.20	12.42	13.10
Crude protein	38.24	38.13	38.19
Crude lipid	9.13	9.11	9.20

^a^ Vitamin premix (per 100 g premix): retinol acetate, 0.043 g; thiamin hydrochloride, 0.15 g; riboflavin, 0.0625 g; Ca pantothenate, 0.3 g; niacin, 0.3 g; pyridoxine hydrochloride, 0.225 g; para-aminobenzoic acid, 0.1 g; ascorbic acid, 0.5 g; biotin, 0.005 g; folic acid, 0.025 g; cholecalciferol, 0.0075 g; α-tocopherol acetate, 0.5 g; menadione, 0.05 g; inositol, 1 g. All ingredients were filled with α-cellulose to 100 g [29]. ^b^ Mineral premix (per 100 g premix): KH_2_PO_4_, 21.5 g; NaH_2_PO_4_, 10.0 g; Ca(H_2_PO_4_)_2_, 26.5 g; CaCO_3_, 10.5 g; KCl, 2.8 g; MgSO_4_·7H_2_O, 10.0 g; AlCl_3_·6H_2_O, 0.024 g; ZnSO_4_·7H_2_O, 0.476 g; MnSO_4_·6H_2_O, 0.143 g; KI, 0.023 g; CuCl_2_·2H_2_O, 0.015 g; CoCl_2_·6H_2_O, 0.14 g; calcium lactate, 16.50 g; Fe-citrate, 1 g. All glucose was diluted with α-cellulose to 100 g [29].

**Table 2:** Amino acid composition of diets (% dry matter).

Amino Acid	Experimental Diets		
	LM	MM	HM
Asparagine	3.42	3.60	3.55
Threonine	1.40	1.43	1.44
Serine	1.65	1.71	1.73
Glutamic acid	6.86	6.99	7.05
Glycine	3.09	2.48	1.77
Alanine	1.99	2.04	2.03
Cystine	0.46	0.45	0.47
Valine	1.63	1.67	1.67
Methionine	0.48	1.05	1.72
Isoleucine	1.45	1.48	1.49
Leucine	3.17	3.23	3.20
Tyrosine	1.15	1.23	1.18
Phenylalanine	1.86	1.93	1.86
Lysine	2.35	2.35	2.35
Histidine	0.92	0.96	0.95
Arginine	2.60	2.60	2.60
Proline	2.11	2.22	2.16

**Table 3:** Primer pair sequences for quantitative real-time PCR in this study.

Gene	Position	Primer Sequence	Product Size (bp)
*srebp-1*	Forward	TCTTCACACCCTCTGGACGC	162
	Reverse	CCAAGGTTGTAATGGCACGC	
*elovl6*	Forward	TGAGAAGCGGCAATGGATGAAG	164
	Reverse	TGGAGAAGAGGGCCAGGAAGAC	
*fas*	Forward	GTCCCTTCTTCTACGCCATCC	127
	Reverse	CGCTCTCCAGGTCAATCTTCAC	
*cpt-1* *α*	Forward	CATCTGGACACCCACCTCCA	183
	Reverse	ATCTCCTCACCCGGCACTCT	
*caat*	Forward	CATCAAGAGCCAGGAGCCCA	172
	Reverse	CTTCAACAGCAGCCCGCAAA	
*mttp*	Forward	TAGGACAAGCAGGACTTTCCTCA	138
	Reverse	CCACATCCACAAACACATCAACA	
*hsp90*	Forward	TCACCAACGACTGGGAGGAT	83
	Reverse	CAGGAAGAGGAGTGCCCTGA	
*tlr2*	Forward	CATACCAGGACGACGAAC	135
	Reverse	AGACATTGAGCGAGGAGA	
*myd88*	Forward	GCCATCGCAGTCGCCAAGTT	148
	Reverse	GGCATCCTGTTCATCCAGTTCTGAC	
*dorsal*	Forward	CGTCAGCAGCACAGCAGAGAAT	272
	Reverse	CCCGTATTTCCTCCCTCAACTTCAG	
*tube*	Forward	ATTGTGCTGCTGGAGTTGCTGAC	207
	Reverse	CATCGTCGGTCGCTTCTTCTTGG	
*caspase8*	Forward	CATGGTGATGAGAATGAC	120
	Reverse	TTGGATGAAGTAGAGACG	
*caspase3*	Forward	AGGAAAAGTTCACGCCGCTA	103
	Reverse	GGCTGCCTTCTGTCAGGATT	
*bax*	Forward	AGAGATGAAGCAGACCACGC	106
	Reverse	TTCTACGGTGGGTGAGTCCA	
*bcl-2*	Forward	CATCATCTCCCTCTTCGCGG	100
	Reverse	CAGTCCCATCACGTCGATCA	
*β-actin*	Forward	TCGTGCGAGACATCAAGGAAA	178
	Reverse	AGGAAGGAAGGCTGGAAGAGTG	

*Srebp-1*, sterol-regulatory element binding protein 1; *elovl6*, elongase of very-long-chain fatty acids 6; *fas*, fatty acid synthase; *cpt-1α*, carnitine palmitoyl transterase 1*α*; *caat*, carnitine acetyltransferase; mttp, microsomal triglyceride transfer protein; hsp90, heat shock protein 90; *tlr2*, toll-like receptor 2; *myd88*, myeloid differentiation factor 88, *caspase3*, cysteine–aspartic acid protease 3; *caspase8*, cysteine–aspartic acid protease 8; *bax*, B-cell lymphoma-2-associated X; *bcl-2*, B-cell lymphoma-2.

**Table 4:** The growth performance of juvenile *E. sinensis* fed the experimental diets at different water temperatures and dietary methionine levels (mean ± SEM) (*n* = 4 replicate tanks).

Parameters	Experiment Diets						Two-Way ANOVA (*p* Value)		
	NT-LM	NT-MM	NT-HM	HT-LM	HT-MM	HT-HM	TL	ML	TL × ML
WG (%)	147.56 ± 6.63 ^a,^*	184.43 ± 12.3 ^b^	158.31 ± 6.95 ^ab^	215.87 ± 11.28 ^AB,^*	247.65 ± 22.47 ^B^	181.08 ± 5.69 ^A^	0.001	0.007	0.170
SGR (%/day)	2.52 ± 0.09 ^a,^*	3.18 ± 0.17 ^b^	2.70 ± 0.1 ^a^	3.64 ± 0.18 ^AB,^*	4.22 ± 0.38 ^B^	3.04 ± 0.09 ^A^	0.001	0.003	0.139
Survival (%)	79.05 ± 0.9 ^a,^*	91.43 ± 4.36 ^b,^*	80.95 ± 3.43 ^ab^	54.17 ± 6.34 ^A,^*	52.08 ± 4.54 ^A,^*	75.71 ± 0.71 ^B^	0.001	0.036	0.003
MR (%)	106.67 ± 3.43 ^a^	120.95 ± 2.52 ^b^	105.71 ± 4.95 ^a^	120.95 ± 5.79 ^AB^	127.62 ± 3.43 ^B^	107.62 ± 2.52 ^A^	0.036	0.003	0.324
FI (g)	1.29 ± 0.03 ^a,^*	1.30 ± 0.02 ^a,^*	1.29 ± 0.02 ^a,^*	1.58 ± 0.01 ^B,^*	1.62 ± 0.01 ^B,^*	1.52 ± 0.02 ^A,^*	0.001	0.028	0.087
FCR (%)	1.3 ± 0.03 ^b,^*	1.16 ± 0.02 ^a^	1.21 ± 0.06 ^ab^	1.11 ± 0.04 ^A,^*	1.15 ± 0.02 ^AB^	1.24 ± 0.02 ^B^	0.069	0.061	0.003
PER (%)	2.01 ± 0.03 ^a,^*	2.44 ± 0.01 ^c^	2.29 ± 0.01 ^b^	2.58 ± 0.01 ^C,^*	2.48 ± 0.01 ^B^	2.23 ± 0.02 ^A^	0.002	0.001	0.001

NT-LM, crabs fed a low-dietary-methionine level diet at 24 °C; NT-MM, crabs fed a medium-dietary-methionine level diet at 24 °C; NT-HM, crabs fed a high-dietary-methionine level diet at 24 °C; HT-LM, crabs fed a low-dietary-methionine level diet at 30 °C; HT-MM, crabs fed a medium-dietary-methionine level diet at 30 °C; HT-HM, crabs fed a high-dietary-methionine level diet at 30 °C. Means with ^a^, ^b^ and ^c^ are significant differences in different dietary methionine level diets at 24 °C. Means with ^A^, ^B^ and ^C^ are significant differences in different dietary methionine level diets at 30 °C. Means with * are significantly different in different water temperatures at the same dietary methionine level diets. (1) Weight gain (%) = (Wt − W0)/W0 × 100. (2) Specific growth rate (% day^−1^) = [lnWt − ln W0]/T × 100. (3) Survival (%) = Nf/Ni × 100. (4) Molting frequency rate = 100 × Nm/Nf. (5) Feed intake (g) = Total feed weight/final number. (6) Feed conversion ratio = Fi/(Wt − W0 + Wd). (7) Protein efficiency ratio = (W t- W0)/Fi × Pi. Wt, W0 and Wd are the means of the final wet body weight, initial wet body weight and dead crab weight, respectively. Nf, Ni, and Nm are the final crab number, initial crab number and molting number, respectively. T and Fi are the duration (days) of the experiment and feed intake, respectively, and Pi is the feed protein content (dry weight).

**Table 5:** Proximate composition of juvenile *E. sinensis* fed different experimental diets (mean ± SEM) (*n* = 4 replicate tanks).

Parameters	Experiment Diets						Two-Way ANOVA(*p* Value)		
	NT-LM	NT-MM	NT-HM	HT-LM	HT-MM	HT-HM	TL	ML	TL × ML
Moisture (%)	67.83 ± 0.77	68.16 ± 0.68	64.8 ± 1.89	69.33 ± 3.11	68.85 ± 1.71	65.66 ± 1.79	0.512	0.165	0.973
Ash (%)	12.21 ± 0.07	12.09 ± 0.04	12.19 ± 0.08	12.14 ± 0.07	12.27 ± 0.05	12.21 ± 0.11	0.469	0.911	0.267
Crude protein (%)	11.95 ± 0.36 ^a^	12.94 ± 0.2 ^ab,^*	14.01 ± 0.48 ^b^	10.74 ± 0.37 ^A^	10.8 ± 0.41 ^A,^*	13.06 ± 0.59 ^B^	0.001	0.001	0.357
Crude lipid (%)	3.66 ± 0.23 ^a,^*	4.17 ± 0.31 ^ab,^*	4.98 ± 0.32 ^b^	4.78 ± 0.18 *	5.11 ± 0.08 *	5.01 ± 0.39	0.008	0.045	0.143

NT-LM, crabs fed a low-dietary-methionine level diet at 24 °C; NT-MM, crabs fed a medium-dietary-methionine level diet at 24 °C; NT-HM, crabs fed a high-dietary-methionine level diet at 24 °C; HT-LM, crabs fed a low-dietary-methionine level diet at 30 °C; HT-MM, crabs fed a medium-dietary-methionine level diet at 30 °C; HT-HM, crabs fed a high-dietary-methionine level diet at 30 °C. Means with ^a^, ^b^ are significant differences in different dietary methionine level diets at 24 °C. Means with ^A^, ^B^ are significant differences in different dietary methionine level diets at 30 °C. Means with * are significantly different in different water temperatures at the same dietary methionine level diets.

## Data Availability

The data presented in this study are available on request from the corresponding author. The data are not publicly available as they contain information that could compromise the privacy of research participants.
